# Characteristic Video Laryngeal Endoscopic "Pharyngeal Rotation" in Unilateral Pharyngeal Constrictor Muscle Paresis: A Case of Herpes Zoster Pharyngitis

**DOI:** 10.7759/cureus.51781

**Published:** 2024-01-07

**Authors:** Megumi Sone, Daisuke Mizokami, Saki Takihata, Akihiro Shiotani, Koji Araki

**Affiliations:** 1 Otolaryngology-Head and Neck Surgery, National Defense Medical College, Tokorozawa, JPN; 2 Otolaryngology-Head and Neck Surgery, Nishisaitama Chuo National Hospital, Tokorozawa, JPN

**Keywords:** varicella zoster virus, videoendoscopic evaluation of swallowing, pharyngeal rotation, glossopharyngeal nerve palsy, herpes zoster pharyngitis

## Abstract

Herpes zoster pharyngitis (HZP) is a rare condition that should be considered as a differential diagnosis of acute dysphagia secondary to unilateral glossopharyngeal and/or vagal nerve palsy. Although early treatment is important to avoid adverse sequelae, serological diagnosis of varicella zoster virus (VZV) takes over a few days. Therefore, it is important to actively suspect VZV infection based on physical findings. Mucocutaneous lesions, curtain signs, and laryngeal palsy are well-known characteristic physical findings. In addition to these findings, the video laryngeal endoscopic finding that the pharyngeal constrictor muscles contract on only one side during swallowing, showing an appearance of "pharyngeal rotation", is one of the characteristic findings of glossopharyngeal/vagal nerve palsy and can support the diagnosis.

We report the case of an 82-year-old Asian female who presented with acute dysphagia, sore throat, left ear pain, and fever that persisted for several days. Initial video laryngeal endoscopy revealed a markedly decreased pharyngeal reflex and significant salivary retention without mucosal vesicular lesions. Repeat videoendoscopic evaluation of swallowing revealed characteristic pharyngeal rotation, which was helpful in diagnosing unilateral pharyngeal constrictor muscle paresis, thus suggesting unilateral glossopharyngeal/vagal nerve palsy. An increase in serum antibody titers (IgG and IgM) against VZV was observed. Bilateral differences and rotation of the pharynx during pharyngeal contraction can be detected endoscopically in pharyngeal constrictor muscle paresis caused by glossopharyngeal/vagal nerve palsy and should be evaluated during video laryngeal endoscopy in patients with dysphagia.

## Introduction

Varicella zoster virus (VZV) is known to cause chicken pox, shingles, and Ramsay Hunt syndrome; however, it can also cause acute dysphagia [1]. Pharyngitis caused by herpes zoster virus (herpes zoster pharyngitis (HZP)) is a rare disorder that causes acute dysphagia secondary to unilateral glossopharyngeal and/or vagal nerve palsy caused by VZV infection [1-3]. The clinical prognosis is difficult, and long-term sequelae can persist in many cases. Nisa et al. reported that, based on 38 references and a literature review of 54 cases of pharyngolaryngeal involvement by VZV, 78% had odynophagia and 17% had pharyngeal paresis, which causes swallowing impairment [1].

Currently, polymerase chain reaction (PCR) and serological testing, including enzyme-linked immunosorbent assay (ELISA), viral culture, and direct fluorescent antigen (DFA), are the main methods for detecting VZV [4,5]. However, laboratory studies are time-consuming in many hospitals if there are no vesicles or skin lesions to collect samples from. To begin with, we cannot perform these tests without a suspected VZV infection. Therefore, it is important to actively suspect VZV infection based on physical findings at the early stage; however, typical mucocutaneous lesions, uvula deviation, and vocal cord paresis are not always observed [1,6]. In such cases, the video laryngeal endoscopic finding that the pharyngeal constrictor muscles contract on only one side during swallowing, showing an appearance of "pharyngeal rotation", is one of the characteristic findings of glossopharyngeal/vagal nerve palsy including HZP and can support the diagnosis. This finding is known to some otolaryngologists but has not yet been officially reported.

In this particular case report, we present a characteristic video of unilateral pharyngeal constrictor muscle paresis and describe its detailed mechanisms.

## Case presentation

**Figure 1:**
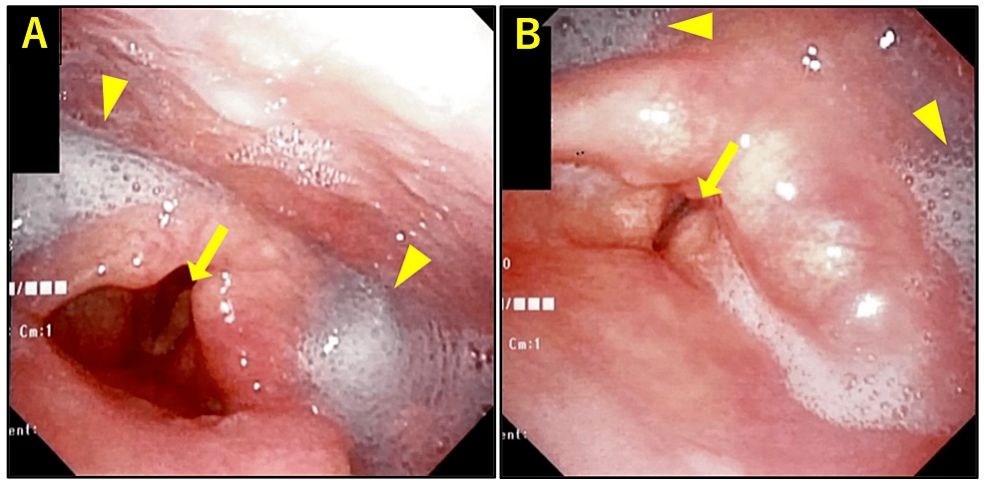
Laryngeal endoscopic images at the first visit. A) While breathing. B) While speaking. Arrows indicate incomplete left vocal cord paralysis. Arrowheads indicate significant pooling of saliva. No mucosal vesicular lesions were observed.

An 82-year-old woman presented with severe dysphagia, sore throat, left ear pain, and fever for six days. She didn't have a spillover or cough. Physical examination revealed no mucosal vesicle lesions or facial, tongue, shoulder, or palatal muscle paralyses. Initial video laryngeal endoscopy revealed markedly decreased pharyngeal reflexes, which were induced only once every few times, significant pooling of saliva, and incomplete left vocal cord paralysis (Figure 1). 

Laboratory tests revealed a white blood cell count of 12300/mm^3^ with 77.9% neutrophils and 12.6% lymphocytes. Serum C-reactive protein level was 1.91 (<0.3) mg/dL. Chest radiography did not reveal any abnormalities; however, plain chest computed tomography (CT) showed an infiltrating shadow suggestive of mild aspiration pneumonia. Magnetic resonance imaging (MRI) of the central nervous system revealed no abnormalities. Antimicrobial therapy for mild aspiration pneumonia, nasogastric tube feeding, and swallowing rehabilitation were initiated.

Videoendoscopic evaluation of swallowing revealed a characteristic finding of pharyngeal constrictor muscle paresis, in which the muscles contracted on only one side during swallowing and the pharynx demonstrated a counterclockwise rotation (Video 1).

**Video 1:** "Pharyngeal rotation" in unilateral glossopharyngeal/vagal nerve palsy (herpes zoster pharyngitis).

Eight days after admission, the serum titers of antibodies against VZV turned out to be as follows: IgG >128 (negative, <2.0) and IgM 6.39 (negative, <0.80). On the same day, one week of oral valacyclovir and corticosteroids were added to the treatment.

Four weeks later, the patient was able to start swallowing rehabilitation and transferred to a rehabilitation hospital. Four months later, the patient was able to feed orally and was discharged from the hospital.

## Discussion

In the present case, the patient complaining of dysphagia was diagnosed with HZP, and "pharyngeal rotation" on video laryngeal endoscopy due to unilateral pharyngeal constrictor muscle paresis helped in the diagnosis.

VZV is known to cause chicken pox, shingles, and Ramsay Hunt syndrome; however, it can also cause glossopharyngeal and/or vagal nerve palsy as a cranial neuropathy, thus resulting in acute dysphagia [2]. VZV reactivation in the vagal ganglia is thought to cause acute dysphagia. Based on prevalence studies in 18 randomly selected fresh cadaver heads, Vrabec and Payne reported that VZV is more likely to be detected in the vagal ganglia than in the geniculate ganglia [7]. Cases of acute dysphagia due to VZV reactivation, as in the present case, may be more common than previously reported.

The glossopharyngeal and vagal nerves have a close anatomical and functional relationship [1]. Both nerves originate from the medulla oblongata, transverse "pars nervosa" of the anterior part of the jugular foramen, and provide branches to the pharyngeal plexus and innervate pharyngeal constrictor muscles [8,9]. Intracranial neural interconnections of the glossopharyngeal and vagal nerves before the "pars nervosa" are rare. Tubbs et al. reported that in 40 adult human cadavers (80 sides), the incidence of intracranial neural connections between the glossopharyngeal and vagal nerves was 2.5% [8]. Extracranially, examples of neural communication are between the inferior or Andersch's ganglion (petrous ganglion, inferior ganglion) of CN IX (glossopharyngeal nerve), the jugular ganglion (superior ganglion) of CN X (vagal nerve), and the glossopharyngeopneumogastric or van Haller's ansa, formed between the contributions from CN IX and the auricular branch of the vagal nerve (Arnold's nerve) [9]. Subsequently, they send branches to the pharyngeal plexus and innervate the pharyngeal constrictor muscles through sympathetic innervation of the superior cervical ganglion (SCG).

Mucocutaneous lesions are also characteristic findings; however, based on 38 references and a literature review of 54 cases, Nisa et al. reported that 34% of patients with pharyngolaryngeal involvement caused by VZV did not have a mucocutaneous rash [1].

At the first visit, the current patient presented with a sore throat, left ear pain, and acute dysphagia. She had no mucocutaneous lesions, uvula deviation, or asymmetric movement of the soft palate. Video laryngeal endoscopy revealed a markedly decreased pharyngeal reflex, significant salivary retention, and incomplete left vocal cord paralysis. Hypopharyngeal mucosal lesions were not observed, although salivary retention may have prevented sufficient observation. Repeated videoendoscopic evaluation of swallowing revealed a characteristic left-right difference and rotation of the pharyngeal contraction, which helped diagnose unilateral pharyngeal constrictor muscle palsy and HZP.

**Figure 2:**
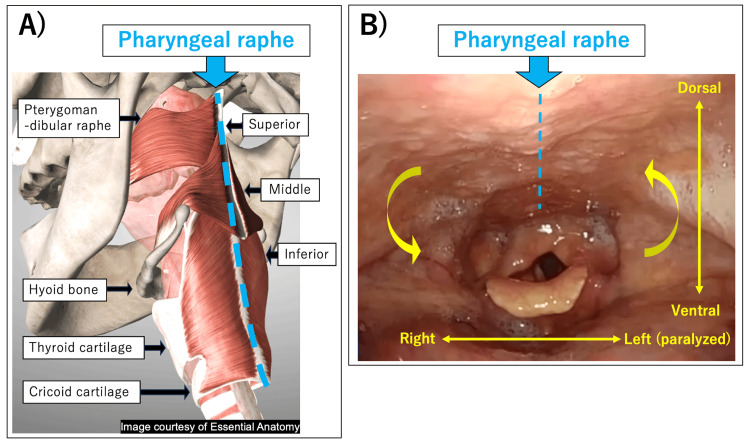
Mechanism of "pharyngeal rotation" in unilateral pharyngeal constrictor muscle paresis. A) Anatomy of pharyngeal constrictor muscles (superior/middle/inferior). Ventrally, the pharyngeal constrictor muscles are anchored to the pterygomandibular raphe, hyoid bone, and cricoid/thyroid cartilage. Dorsally, they are attached to each other in the movable pharyngeal raphe (Image courtesy of Essential Anatomy. Thanks to @3D4Medical). B) A case of left pharyngeal constrictor muscle paresis. The paralyzed constrictor muscles (left) are pulled dorsally to the non-paralyzed side (right) at the pharyngeal raphe, which causes rotation.

The mechanism of "pharyngeal rotation" can be similar to that of the uvula deviation and the asymmetric movement of the soft palate. Both sides of the pharyngeal constrictor muscles are anchored ventrally to the pterygomandibular raphe, hyoid bone, and cricoid/thyroid cartilage. Dorsally, they are attached to each other in a movable pharyngeal raphe. Therefore, if the left side of the constrictor muscles paralyzes while swallowing, only the right constrictor muscles constrict, and the pharyngeal raphe is pulled to the right side, causing a counterclockwise rotation of the pharynx (Figure 2).

As a limitation, the motion of the soft palate may cause the camera to move. However, if the pharynx rotates unaccompanied by laryngeal motion, the pharyngeal rotation can be understood to be caused by pharyngeal constrictor muscle paresis.

The muscles that elevate the soft palate are the levator veli palatini muscle (LVP) and the tensor veli palatini muscle (TVP). Since the TVP is innervated by CN V3, the LVP is the muscle that raises the soft palate and is associated with glossopharyngeal/vagal nerve palsy [10]. The innervation of the LVP in adults is controversial, but Shimokawa et al. dissected 50 head halves of 25 cadavers and reported that 8% were innervated by the glossopharyngeal nerve, 72% by the communicating branches of the glossopharyngeal and vagal nerves, and 20% by the vagal nerve [11]. Mu et al. reported that, in six cadavers, the LVP were innervated by the vagal nerve and branches of the lesser palatini nerve [10]. In any case, there are individual differences in the innervation of the LVP, with a tendency of glossopharyngeal < vagal nerve. 

In the present case, it is possible the glossopharyngeal nerve palsy was more prominent than the vagal nerve palsy; therefore, the soft palate was not paralyzed. It can be supported by the fact that this patient had severe dysphagia but only minor vocal cord paralysis.

Currently, PCR and serological testing, including ELISA, viral culture, and DFA, are the main methods for detecting VZV [4,5]. However, laboratory studies are time-consuming in many hospitals if there are no vesicles or skin lesions to obtain tissues. First, we must actively suspect VZV infection for testing.

For detecting VZV infection, other than serological tests (e.g., ELISA) performed in this case, PCR, viral culture, and DFA are the main tests [4,5]. In the presence of vesicular lesions, DFA provides rapid results. Viral culture is time-consuming and usually requires vesicular lesions [4]. PCR also usually requires vesicle lesions but can provide results within a day in facilities that have access. However, many facilities outsource the tests; consequently, the results take longer. PCR can also be performed on the cerebrospinal fluid (CSF), but the positivity rate is not high in cases presenting with lower cranial nerve symptoms. In 2023, Murakami et al. summarized previously reported VZV infection cases with lower cranial polyneuropathy other than VII and VIII, in which CSF VZV-DNA PCR testing was performed [12]. According to this report, out of 11 cases that had no meningeal signs, only four cases tested positive on CSF VZV-DNA PCR.

Therefore, reactivation is typically diagnosed by serological tests in the absence of mucosal or skin lesions. Although these methods are not usually useful for early diagnosis, Sauerbrei et al. reported that only 48.2% of patients were infected with VZV in the first serological test (range, 3-8 days); however, 85.7% had a second recovery serum sample with markedly elevated IgG, IgA, or IgM [13]. Furthermore, completing the first serum test requires more than a few days.

In cases of VZV cranial palsy in the early stages, it is necessary to suppress the spread of the virus and reduce the vicious cycle of edema, constriction, and ischemia by administering antiviral therapy and steroids [14]. Therefore, it is important to suspect a VZV infection based on physical findings and initiate treatment as soon as possible. Mucosal vesicle lesions are characteristic of VZV infection but are not always present. Sore throat and ear pain are not specific findings. Uvula deviation and vocal cord palsy are not always observed.

The video laryngeal endoscopic findings shown in Video 1, in which the pharynx shows a counterclockwise rotation due to the bilateral difference in pharyngeal contraction, can help in the diagnosis of pharyngeal constrictor muscle paresis due to glossopharyngeal and/or vagal nerve palsy. This finding can be easily observed as part of routine video laryngeal endoscopy with low invasiveness and can be repeated. When performing video laryngeal endoscopy in patients with dysphagia, "pharyngeal rotation" while swallowing is an easy and helpful finding in diagnosing unilateral pharyngeal constrictor muscle paresis, including HZP.

## Conclusions

We reported a case of HZP with characteristic "pharyngeal rotation" on laryngeal endoscopy in which the pharyngeal constrictor muscles contracted on only one side during swallowing, supporting the diagnosis of glossopharyngeal and/or vagal nerve palsy. Although this finding is known to some otolaryngologists, it has not been officially reported. It can be easily observed as a part of routine video laryngeal endoscopy with low invasiveness. Video laryngeal endoscopy in patients with dysphagia and "pharyngeal rotation" while swallowing helps diagnose unilateral pharyngeal constrictor muscle paresis, including HZP, and should be actively observed.
